# Mothers' Milk

**Published:** 1891-01-10

**Authors:** 


### DIETETICS.

### MOTHERS' MILK.

The neglect of the study of the variations in the composition of human milk when contrasted with the labour that has been devoted to the analysis of that of the cow, is far from creditable, for it would be but reasonable that some rapid process of estimating the percentage of the fat, albumen, and sugar, should be considered as incumbent on the physician in all cases in which the mothers' milk appears insufficient or unsuitable for the infant, as the estimation of urea, chlorides, &c., of the urine in diseases of the kidney or derangement of the nutritive processes in the adult. Dr. Rotch, of Boston, U.S., has recently published the results of numerous observations made under various circumstances having a practical bearing on the question of infant feeding. He found the normal average percentage to be in round numbers, fat 4, albuminoids, 1 to 2, sugar, 7, and ash, 0 2; the range within which the infants appeared to thrive being, fat, 3 to 4, albumens, 1 to 3, sugar, 6 to 7, and ash, 0·1 to 0·2. A remarkable result was seen in the effect of too long or too short intervals being allowed to elapse between successive nursings. When shorter the percentage of solids was greater, and when too long, the milk was more watery; thus with intervals of two hours the solids were 15·32, and the water 84·68, with twelve hours they were 10·14 and 89·86 respectively, whence it follows that so far from becoming concentrated by delay, the milk is rendered poorer, while the harm done by too frequent nursing may be due as much to the excessive amounts of solids as to the stomach not having had time to empty itself. Food and exercise naturally influenced the quality of the milk, a liberal diet with little exercise giving a rich milk, provided the mother's digestion were good, but when her sedentary life interfered with the proper assimilation of her food, the albuminoids were in great excess, and the milk disagreed with the infant, causing vomiting, colic, and diarrhœa. Except when the mother was very ill-fed, in fact half-starved, was suffering from exhausting illness, or great mental anxiety, or was again pregnant, the percentage of sugar varied but little; deficient exercise tended to increase the albumen, and the proportion of fat was found to depend directly on the amount of albumen in the food. Dr. Rotch does not refer to the views of the Munich School on the genesis of fat in the organism, but these observations of his confirm the conclusion of Erwyn Voit and other distinguished pupils of Prof. Pettenkofer, deduced from accurate and exhaustive experiments, that fat is formed directly from the metabolism of albumen, which after combining with water is broken up into fat and urea, the fat being ordinarily further metabolised into carbonic acid, and water, like the carbohydrates and hydrocarbons of the food, or under other conditions, stored up in the tissues. This storage of fat occurs either when there is little demand for force and heat production, or when a more easily oxidisable fuel, as the carbohydrates, is supplied in abundance. The accumulation of fat that follows a diet containing an excess of carbohydrates, which has caused starch and sugar to be looked on as fat producers, has been generally misinterpreted. They are not transformed into fat, but act as a "spaarmittel," *i.e.*, furnishing a readier fuel, they save the oxidation of the albumen and allow the fat formed from it to accumulate instead of being burned off. Analysis of the milk of a healthy wet nurse during three consecutive periods of a month each, of poor diet, rich food with little exercise, and of good diet and healthy exercise indoors and out, illustrate all these effects very clearly.

| | Period I. | II. | III. |
|---|---|---|---|
| Fat ... ... ... ... | 0·72 | 5·44 | 5·50 |
| Albumens ... ... ... | 2·53 | 4·61 | 2·90 |
| Sugar... ... ... ... | 6·75 | 6 25 | 6·60 |
| Ash ... ... ... ... | 0·22 | 0·20 | 0·14 |
| Total solids ... ... ... | 10·22 | 16·50 | 15 15 |
| Water ... ... ... | 39·78 | 83·50 | 84 86 |

The milk of the first period was marked by want of fat, owing to an insufficient amount of meat, and that of the second produced gastro intestinal derangements in the infant from the excess of albumen consequent on defective metabolism. As with the milk of cows, the greatest variation was observed in the percentage of fat, viz.: from 0·72 to 5·50, or as 1 to 7, while the albumen varied from 2·40 to 4·61, or nearly as 1·2, according to the amount of exercise taken in proportion to the diet. When, in the absence of analysis, the habits of the mother are such as to afford a presumption that the disturbance of the infant's digestion is due to an excess of albumen in the milk, active out-door exercise should be enjoined on her, or, if that be impossible, some water might be given to the infant after each application to the breast.

